# Nanostructured Ge and GeSn films by high-pressure He plasma sputtering for high-capacity Li ion battery anodes

**DOI:** 10.1038/s41598-022-05579-z

**Published:** 2022-02-02

**Authors:** Giichiro Uchida, Kenta Nagai, Yuma Habu, Junki Hayashi, Yumiko Ikebe, Mineo Hiramatsu, Ryota Narishige, Naho Itagaki, Masaharu Shiratani, Yuichi Setsuhara

**Affiliations:** 1 Faculty of Science and Technology, Meijo University, 1-501 Shiogamaguchi, Tempaku-ku, Nagoya, 468-8502 Japan; 2 Graduate School and Faculty of Information Science and Electrical Engineering, Kyushu University, 744 Motooka, Nishi-ku, Fukuoka, 819-0395 Japan; 3 Joining and Welding Research Institute, Osaka University, 11-1 Mihogaoka, Ibaraki, 567-0047 Japan

**Keywords:** Batteries, Electrical and electronic engineering, Synthesis and processing

## Abstract

We fabricated nanostructured Ge and GeSn films using He radio-frequency magnetron plasma sputtering deposition. Monodisperse amorphous Ge and GeSn nanoparticles of 30–40 nm size were arranged without aggregation by off-axis sputtering deposition in the high He-gas-pressure range of 0.1 Torr. The Ge film porosity was over 30%. We tested the charge/discharge cycle performance of Li-ion batteries with nanostructured Ge and GeSn anodes. The Ge anode with a dispersed arrangement of nanoparticles showed a Li-storage capacity of 565 mAh/g after the 60th cycle. The capacity retention was markedly improved by the addition of 3 at% Sn in Ge anode. The GeSn anode (3 at% Sn) achieved a higher capacity of 1128 mAh/g after 60 cycles with 92% capacity retention. Precise control of the nano-morphology and electrical characteristics by a single step procedure using low temperature plasma is effective for stable cycling of high-capacity Ge anodes.

## Introduction

The automobile industry is currently shifting towards hybrid and electric vehicles powered by electrochemical energy storage systems, or batteries. However, these batteries are less fuel efficient than conventional gasoline systems, and it is therefore important to develop high-performance batteries that have a high energy density, high electromotive force, and a long charge/discharge cycle life. Recently, because of the limited capacity of carbon (graphite) anodes in Li-ion batteries, the development of alternative anode materials that are reactive with Li has been actively promoted^1–3^. Among these, Si^4–10^, Ge^11–20^, and Sn^21,22^ are the most interesting materials because they have high theoretical capacities of 4200, 1600, and 993 mAh/g, respectively, which are much higher than the value of 372 mAh/g for conventional carbon active material. However, high-capacity Si, Ge, and Sn materials undergo large volume changes during Li alloying/de-alloying reactions of 400, 370, and 360%, respectively, and prolonged cycling leads to electrode pulverization from the current collector, resulting in capacity fading. In this study, we focus on Ge as a Li-ion battery anode material, for which the room temperature diffusivity of Li is 400 and 40 times higher than that for Si and Sn, respectively^12,23–25^, and the intrinsic electrical conductivity σ is the order of 1 S/m which is four orders of magnitude higher than that of Si at room temperature of 10^−4^ S/m. The significant difference in σ is mainly due to the variation of carrier density, which is related to the electron density in the conduction band. Ge has a smaller band gap *E*_g_ of 0.67 eV and a higher electron density in the conduction band than Si with *E*_g_ of 1.1 eV.

Various approaches have been used to enhance the cycling stability of high-capacity Ge anodes. These include different morphologies such as nanoparticles^11,16,17^, nanowires^12,14,18–20^, and nanoporous structures^15^, which are expected to show better structural stability against volume changes.

Lee et al. reported that amorphous Ge nanoparticles, which were produced by a GeCl_4_ chemical solution process, exhibited high Li-storage capacity over 1450 mAh/g after 19 cycles^11^. Cho et al. showed that GeSn alloy nanoparticles, synthesized by a laser photolysis process using vapor Ge(CH_3_)_4_ and Sn(CH_3_)_4_, led to a high capacity of 1010 mAh/g after 50 cycles in a Li ion battery^17^. However, the anode film formation procedure using nanoparticles is complex and consists of many steps. The synthesized nanoparticles are mixed with a polymer binder, carbon black, and N-methylpyrrlidone (NMP) as a solvent. Then, the nanoparticle slurry is coated on metal foils for current collectors and finally sintered for rigid film formation. In general, controlling the porosity and nanoparticle aggregation in the nanoparticle film is difficult in the slurry coating process. Particle aggregation is not an ideal anode film structure in Li ion batteries, because the large number of grain boundaries produces a large diffusion resistance of Li ions, as shown schematically by Oh et al.^26^.

1D nanowires have attracted interest due to their ability to accommodate expansion/contraction events related to lithiation/delithiation processes. 1D nanowires can expand both longitudinally and radially, and alleviate the tensile stress that causes cracking in anode materials^18,19^. Mullane et al. fabricated Ge nanowire anodes in a bottom-up process, directly grown on a substrate using a chemical reaction through thermal decomposition of the organometallic Ge precursor diphenylgermane (C_12_H_12_Ge)^18^. Li ion battery cells with Ge nanowire anodes showed a specific capacity of 1,091 mAh/g after 400 cycles^20^. The chemical solution process involves complicated synthesis routes such as formation of solid catalyst seeds on the substrate, and requires precise control of the process parameters, such as temperature, for 1D structure formation. Refino et al. reported vertical Si nanowire array anodes created in a top-down process by plasma etching^9^. Si nanowire arrays were fabricated by applying circular patterns to Si wafers as a template using photolithography, where an n-type Si wafer with high electrical conductivity was used as the active anode material. A Li ion battery cell with a Si-nanowire-array anode showed 0.25 mAh/cm^−2^ with a low capacity fading of 16.7% after 20 charge/discharge cycles. In the dry etching process, many-step procedures such as spin coating of photoresist, baking, pattern transference in lithography, plasma irradiation, and removal of photoresist, are utilized for the 1D structure formation.

We use an alternative method to fabricate Ge nanostructures films for Li-ion-battery anodes with low-temperature plasma. The advantage of our process is that it allows direct fabrication of nanoparticle films on a current collector without pretreatment and temperature control by employing a simple single-step procedure. The gas-phase plasma process enables us to form a binder-free nanostructure anode film with only active material, such as Ge, which also allows precise control of nanoparticle aggregation in the film^27–30^.

## Results and discussion

### Characteristics of nanostructured Ge films

**Figure 1:**
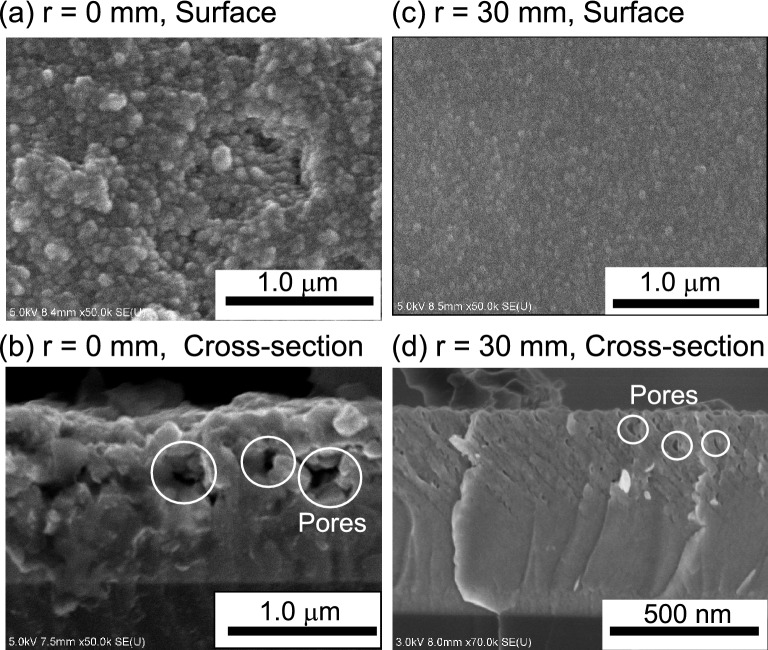
SEM surface and cross-sectional images of Ge films deposited at substrate positions of (**a**,**b**) 0 mm (on-axis position) and (**c**,**d**) 30 mm (off-axis position). The film was deposited at a He gas pressure and flow rate of 0.1 Torr and 15 sccm, respectively.

Figure 1a,b show surface and cross-sectional scanning electron microscopy (SEM) images of films deposited at the center (*r* = 0) of the substrate holder. Ge nanoparticle film was fabricated in a single-step process using low-temperature plasma sputtering under a He-gas high pressure condition of 0.1 Torr. The average size of the nanoparticles was roughly estimated to be 100 nm from the surface SEM by randomly selecting over 20 particles in the SEM image and measuring the particle size using scale-bar software of the SEM.

A porous structure with abundant pores was clearly observed in the film from the cross-sectional SEM image, as shown in Fig. 1b. We evaluated the porosity of the deposited Ge films. We measured the mass density ρ of the deposited films and compared it with the bulk Ge density of 5.32 g/cm^3^. The porosity, calculated as ((5.32 − ρ)/5.32) × 100 (%), was as high as 34%. ρ was estimated from the mass and volume of the deposited Ge nanoparticle film: the film mass was derived from the difference in the substrate mass before and after the deposition process, and the film volume was estimated from the product of the deposition area and the film thickness from the cross-section SEM image.

Figure 1c,d show surface and cross-sectional SEM images of films deposited at 30 mm from the center (*r* = 30 mm) of the substrate holder (off-axis deposition). In this case, the nanoparticles were about half the size, at 56 nm, and the film changed to a more dense structure. It should be emphasized that the porosity of the dense nanoparticle film was still as high as 30%. We also deposited Ge films under an Ar-gas high pressure of 0.1–0.5 Torr^31^. In this case, the film porosity was 13–17%, which was much smaller than the 30–34% observed for the He sputtering film. This indicates that a high He-gas pressure leads to a high-porosity film of Ge nanoparticles.

**Figure 2:**
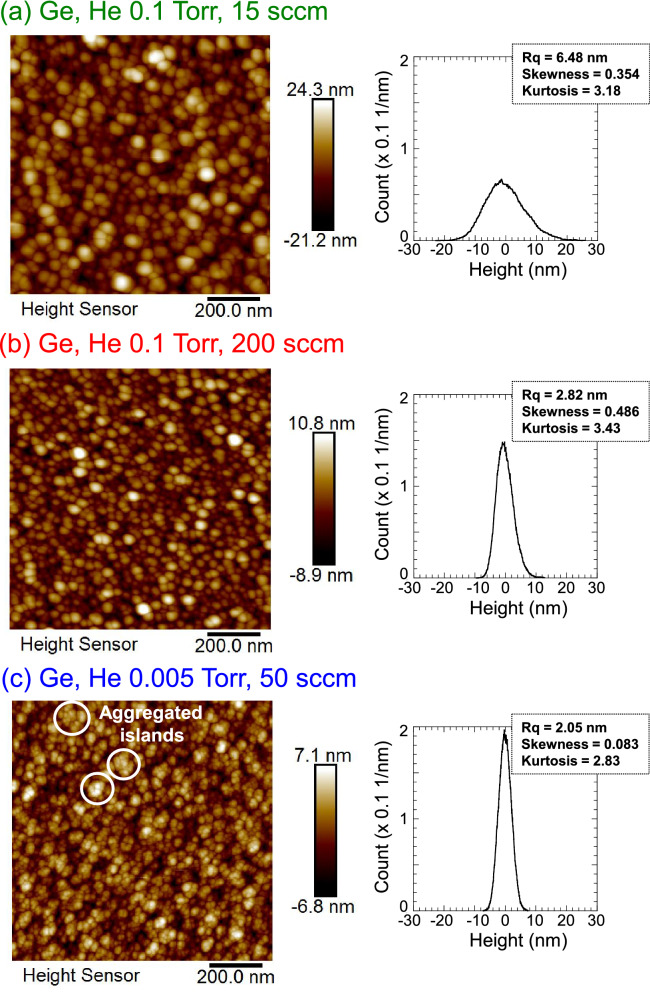
AFM surface image and surface height distribution for Ge film deposited at He gas pressures and gas flow rates of (**a**) 0.1 Torr and 15 sccm, (**b**) 0.1 Torr and 200 sccm, and (**c**) 0.005 Torr and 50 sccm. All Ge films were deposited at substrate positions of 30 mm (off-axis position).

The morphology of the dense nanoparticle films deposited at *r* = 30 mm was analyzed with atomic force microscopy (AFM) in detail. Figure 2a–c show surface images and the surface height distribution for films deposited at He 0.1 Torr and 15 sccm, He 0.1 Torr and 200 sccm, and He 0.005 Torr and 50 sccm, respectively. Ge nanoparticles were separately dispersed over the entire surface for He 0.1 Torr and 15 sccm. The average size of the particles and the surface root-means-square roughness were 41 nm and 6.4 nm, respectively. The size distribution of the nanoparticles was about 10 nm, and an arrangement of relatively monodisperse nanoparticles was realized by plasma off-axis sputtering under a He-gas pressure of 0.1 Torr. The nanoparticle size was controlled by the gas flow rate in the high He-gas pressure sputtering system. When the He gas flow rate was increased to 200 sccm, the particle size and surface root-means-square roughness decreased to 32 nm and 2.8 nm, respectively, and the height distribution of the surface became narrower, as shown in Fig. 2b. We also deposited a film under conventional low-gas-pressure conditions of 0.005 Torr and 50 sccm. As shown in Fig. 2c, many aggregated islands composed of nano-grains could be identified on the film surface, where the average size of the aggregated islands was 35 nm. This demonstrates that the high-pressure condition is effective for forming an arrangement of monodisperse nanoparticles without aggregated islands.

**Figure 3:**
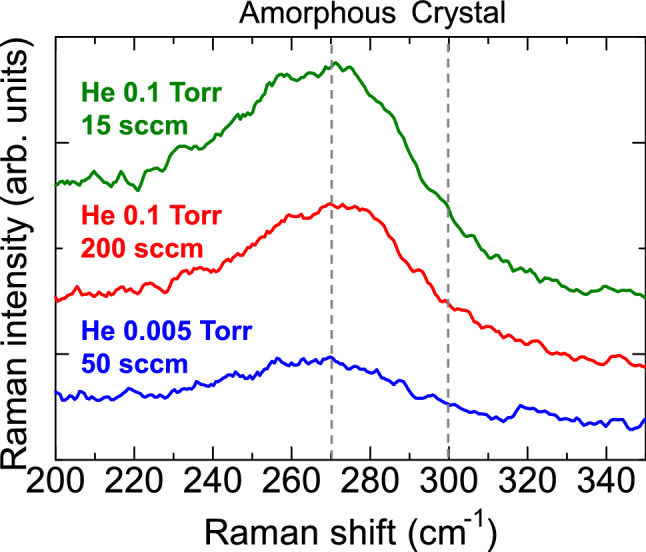
Raman spectra of Ge films deposited at He gas pressures and gas flow rates of 0.1 Torr and 15 sccm, 0.1 Torr and 200 sccm, and 0.005 Torr and 50 sccm. All Ge films were deposited at substrate positions of 30 mm (off-axis position).

Figure 3 shows Raman spectra for Ge films deposited at *r* = 30 mm using different He gas conditions. All the films show a broad peak at 269 cm^−1^, which is attributed to a Ge–Ge mode. The broad peak is assigned to an amorphous structure. The full width at half maximum (FWHM) of the spectra slightly broadened with increasing Ge gas pressure from 0.005 mTorr to 0.1 Torr: the FWHM of the Ge films deposited at He gas pressures and gas flow rates of 0.005 Torr and 50 sccm, 0.1 Torr and 15 sccm, 0.1 Torr and 200 sccm are 44.4, 47.7, and 50.5 cm^−1^, respectively. Such an amorphous structure seems to have more favorable fracture behavior when reacting with Li than a crystalline structure, because amorphization of the crystal structure during lithiation could significantly affect stress generation and fracture, leading to capacity fading^32,33^. Based on the above results, we conclude that high He-gas pressure plasma sputtering in the sub-Torr range gives a dispersed arrangement of amorphous Ge nanoparticles with a high porosity of more than 30%.

**Figure 4:**
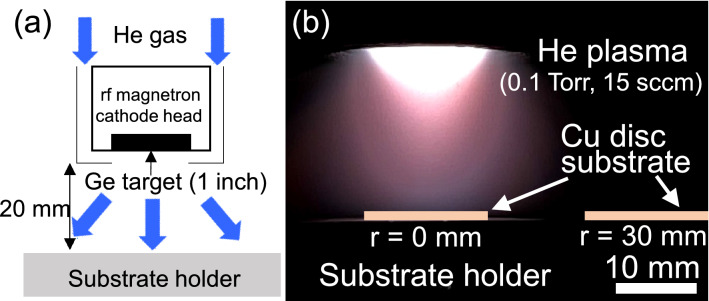
(**a**) Schematic of experimental setup for rf magnetron plasma sputtering and (**b**) image of plasma emission in front of Ge sputtering target.

Godinho et al. reported that He atoms can be incorporated into amorphous Si films during deposition by He plasma sputtering in the conventional mTorr range, resulting in the formation of He bubbles in the film^34^. This is due to the extremely low solubility of He atoms in metals. Porous Si films produced using He sputtering have been applied to Li-ion-battery anodes^6^. The current study differs from previous works with regard to the He gas pressure for sputtering. Our high-porosity Ge film deposited in the sub-Torr range was formed by stacking of nanoparticles, as can be seen from the SEM images in Fig. 1a,b. At a high pressure of 0.1 Torr, Ge nanoparticles could be synthesized in a gas-phase plasma because a higher-density plasma is produced locally in front of the cathode sputtering target (see the image of Fig. 4b), resulting in a large amount of highly reactive excited Ge atoms in the gas phase^35–38^. The shorter collision mean free path for Ge species would cause nucleation and growth of nanoparticles in the gas phase^39,40^, and these nanoparticles would then be transported downstream without aggregation by the neutral gas flow. In addition to the effect of bubble formation in the He sputtering process, the nanoparticle-stacking structure contributes to the observed high porosity of over 30%. Based on the above discussion, nanoparticle production in the sub-Torr high He gas pressure range is an important factor for the fabrication of high porosity film, where the gas flow from the target to substrate helps transport the produced nanoparticles into the substrate. In our experiments, the main difference between nanoparticle films deposited at *r* = 0 and *r* = 30 mm was the particle size: relatively larger particles were deposited on the substrate at *r* = 0, while smaller particles were deposited on the substrate at *r* = 30 mm. This might be due to the difference in residence time of particles in the plasma area for the reaction growth of particles. At *r* = 0, nucleated particles have a relatively long residence time in the plasma area, resulting in the growth of large particles. On the other hand, at *r* = 30 mm, the residence time of particles in the plasma area is rather short, because particles cross the plasma area in the *r* direction to get to the substrate, as understood from Fig. 4a,b. This results in the formation of film composed of smaller 30–40 nm particles.

### Performance of Li ion battery with nanostructured Ge anode

**Figure 5:**
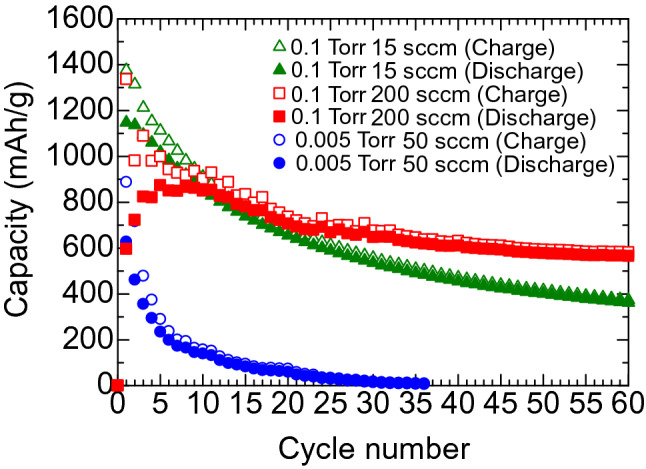
Cycle performance of Li-ion batteries with Ge anodes deposited at He gas pressures and gas flow rate of 0.1 Torr and 15 sccm, 0.1 Torr and 200 sccm, and 0.005 Torr and 50 sccm. All Ge films were deposited at substrate positions of 30 mm (off-axis position).

We tested the performance of Li ion batteries with Ge anodes. Figure 5 shows the gravimetric capacity of Li-ion battery cells with Ge anodes deposited at He gas conditions of 0.1 Torr and 15 sccm, 0.1 Torr and 200 sccm, and 0.005 Torr and 50 sccm, as a function of the number of charge/discharge cycles. The gravimetric capacity was calculated by dividing the observed capacity (mAh) of the Li-ion battery by the mass (g) of the nanostructured Ge film as the active anode material. A high gravimetric capacity of more than 1000 mAh/g was observed for all the test cells in the first cycle. The gravimetric capacity after a few tens of cycles strongly depends on the He gas pressure. For the test cell with a Ge film deposited at 0.005 Torr and 50 sccm, the initial high capacity markedly decreased to 6.7 mAh/g in inverse proportion to the cycle number after 36 cycles. For the test cells with Ge films deposited at 0.1 Torr, the capacity showed a rapid decrease to about 900 mAh/g during the first 10 cycles, and then underwent a gradual decrease. As a result, the test cells with Ge films deposited at 0.1 Torr/15 sccm and 0.1 Torr/200 sccm maintained a capacity of 361 mAh/g and 565 mAh/g after 60 cycles, and showed a capacity retention of 31% and 64%, respectively. We also tested a Li ion battery cell with a Ge anode composed of large particles with sizes of 100 nm, deposited at a substrate position of *r* = 0 under He 0.1 Torr and 15 sccm, as shown in the SEM images of Fig. 1a. The gravimetric capacity rapidly dropped to 6.9 mAh/g after only 3 cycles and showed a poor capacity retention. Our experiment shows that a dispersed arrangement of 30–40-nm nanoparticles with a high porosity over 30% leads to better capacity retention in Li-ion batteries than anode films with aggregated islands.

Nanoparticle films exhibit less crack formation in the anodes of Li-ion battery, as shown schematically by Nitta et al.^2^. It is known that nano-scaling materials facilitate fast charging by increasing the electrode–electrolyte contact area and shortening the diffusion lengths for Li-ion transport^20^. Cheng et al. theoretically suggested that the tensile state of diffusion-induced stress is significantly reduced in magnitude with decreasing particle radius, which can relax the strain in nanoparticles without mechanical fracture^41^. It is also reasonable to suppose that the high porosity contributes to low degradation of the anode material, because the pore spaces act as buffers for volume expansion of Ge grains to several times their original size upon Ge-Li alloying. In addition, the high porosity improves the Li-ion transfer efficiency by allowing easier penetration of the Li-ion electrolyte solution into the anode material. However, regarding the porous effect, the aggregated islands observed in conventional mTorr-range sputtering are not an ideal anode film structure because of the large number of grain boundaries. The large diffusion resistance of Li ions into the interior causes non-uniform volume expansion due to a partial lithiation^26^, resulting in pulverization of the aggregated islands and the related low capacity retention of the Li-ion battery.

### Effects of Sn incorporation in nanostructured Ge anode

**Figure 6:**
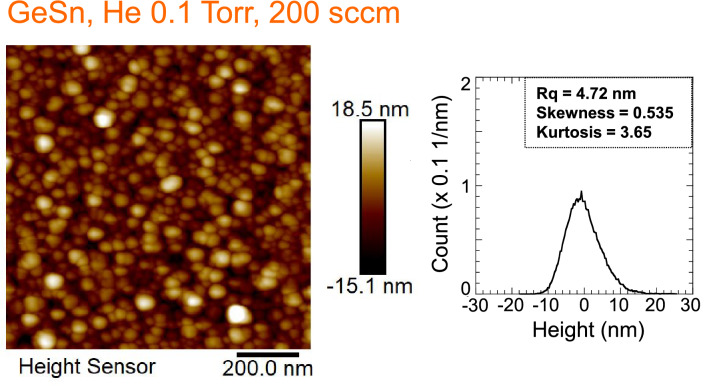
AFM surface image and surface height distribution of GeSn film (3at% Sn) deposited at a He gas pressure and gas flow rate of 0.1 Torr and 200 sccm. The GeSn film was deposited at a substrate position of 30 mm (off-axis position).

**Figure 7:**
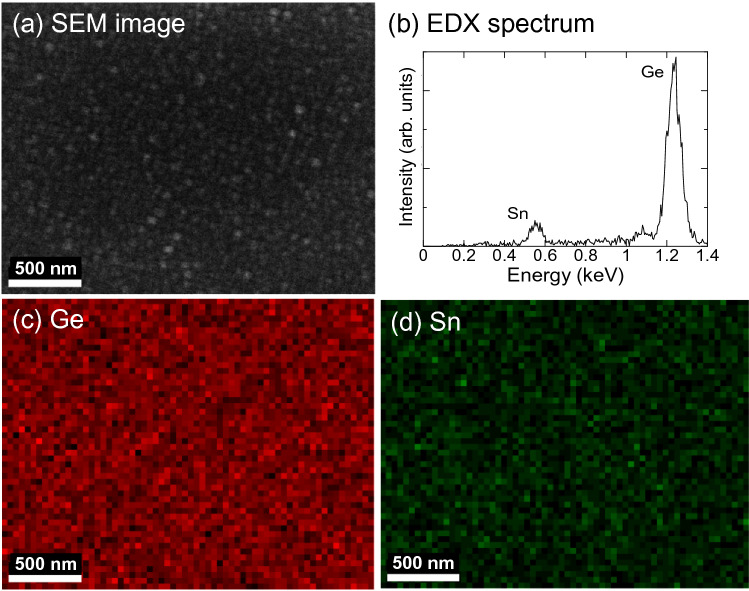
(**a**) SEM surface image and (**b**) SEM–EDX spectra of GeSn film deposited at a He gas pressure and gas flow rate of 0.1 Torr and 200 sccm. SEM–EDX mappings of (**c**) Ge and (**d**) Sn with respect to the area in (**a**). The GeSn film was deposited at a substrate position of 30 mm (off-axis position).

A Li ion battery with a Ge nanostructure anode showed a better capacity of 565 mAh/g after 60 cycles. However, some capacity fading was observed, which might be attributed to the lower electrical conductivity of Ge of 1 S/m. In Li ion batteries, both electrons and Li ions should be transported into the Ge anode for Ge-Li alloying, where the electrochemical reaction of Ge with Li^+^ and e^−^ proceeds at the anode/electrolyte boundary. To improve the electrical conductivity of Ge, an element from the same group IV Sn was added in the Ge film. Metallic Sn has a high electrical conductivity of 10^7^ S/m and a high theoretical capacity 994 mAh/g for Li storage. In our experiment, a GeSn nanostructure film was fabricated using a GeSn sputtering target including 6at% Sn content in Ge. The sputtering conditions are as follows: (1) high He pressure of 0.1 Torr, (2) high gas flow rate of 200 sccm, and (3) off-axis substrate position at *r* = 30 mm. Figure 6 shows an AFM surface image and a surface height distribution for the GeSn film. We observed a nanostructure composed of GeSn nanoparticles with an average size of 34 nm without aggregated islands, with almost the same surface height distribution as that for the Ge nanostructure film in Fig. 2b. A Sn content of 3at% was determined in the GeSn film using energy dispersive X-ray spectroscopy (EDX). There was a slight composition shift between the deposited GeSn film (Sn 3at%) and sputtering target (Sn 6at%), and loss of Sn during sputtering was observed. Generally, in plasma sputtering, a composition shift arises from differences in the sputtering yield, angular flux distribution from the sputtering target, diffusion of sputtered species due to collision with discharge gas atom, and other differences^42^. Fig. 7a–d show an SEM surface image, EDX spectra, and SEM–EDX mappings of (c) Ge and (d) Sn with respect to the area in (a), respectively. The chemical composition is homogeneous and Sn is uniformly distributed without segregation in the Ge film.

**Figure 8:**
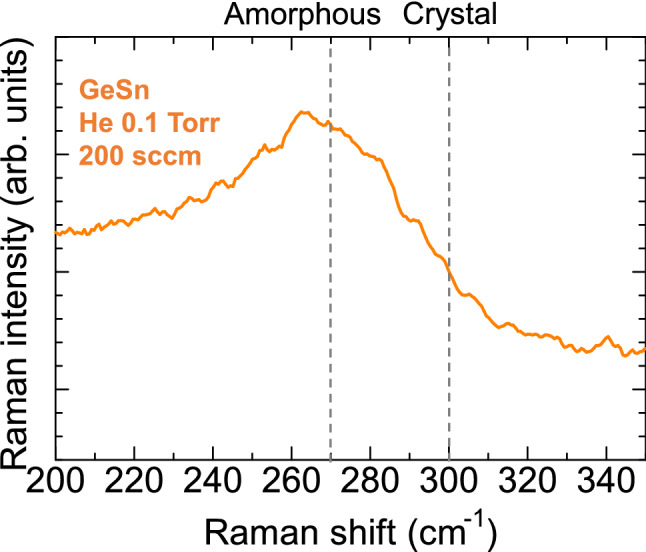
Raman spectra of GeSn film (3at% Sn) deposited at a He gas pressure and gas flow rate of 0.1 Torr and 200 sccm. The GeSn film was deposited at a substrate position of 30 mm (off-axis position).

As can be seen in Fig. 8, the Raman spectrum showed a broad peak at 264 cm^−1^ (FWHM: 57.4 cm^−1^), indicating a small shift to a wavenumber lower than 269 cm^−1^ for amorphous Ge. Zhang et al. reported Raman scattering study of amorphous GeSn films in detail^43^. The broad phonon scattering peak near 270 cm^−1^ shifted to lower wavenumbers with the increase in Sn content. Lieten et al. reported that for amorphous GeSn films with 4.5% and 11.3%, the broad Raman spectrum shifted to lower wavenumbers with respect to the bulk Ge–Ge phonon peak at 301 cm^−1^ by − 28.0 cm^−1^ and − 31.3 cm^−1^, respectively^44^. The peak shift is due to increased disorder in the bond distances of Ge–Ge by the addition of Sn, and suggests the formation of an amorphous GeSn alloy structure^45^.

The solid solubility limit of Sn in Ge is theoretically less than 1%, which is expected for a solid solution in thermal equilibrium^46^. In our study, nanostructured GeSn film with Sn 3at% was fabricated by non-thermal-equilibrium reactions which take place in the gas phase or on the substrate during plasma sputtering. Cho et al. have reported that Ge_0.95_Sn_0.05_ nanoparticles with a single crystalline cubic phase are synthesized in the gas-phase laser photolysis reaction^17^. These results indicate that a non-thermal-equilibrium process realizes synthesis of GeSn alloy with more than 1% content of Sn.

**Figure 9:**
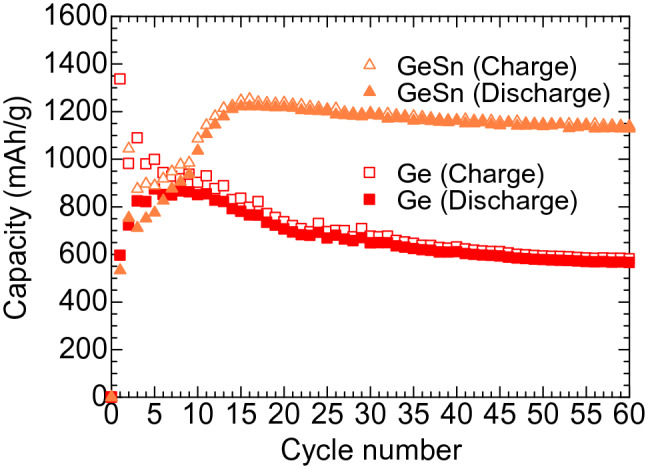
Cycle performance of Li-ion batteries with Ge and GeSn (3at% Sn) anodes deposited at a substrate position of 30 mm under a He gas pressure and gas flow rate of 0.1 Torr and 200 sccm, respectively.

Figure 9 compares the gravimetric capacity of a Li-ion battery cell with a GeSn nanostructure anode to that with a Ge nanostructure anode. Both the GeSn and Ge anodes were fabricated under the same sputtering conditions of He 0.1 Torr, 200 sccm, at *r* = 30 mm. The GeSn battery cell showed stable behavior with a Coulombic efficiency of 99% after the first 14 cycles, where the highest gravimetric capacity of 1221 mAh/g was observed. We also observed little fading of capacity over 50 cycles, and a high capacity of 1128 mAh/g was maintained after 60 cycles, where the capacity retention was as high as 92%. The results show that the high conductivity of a GeSn anode offers a significant advantage for high capacity retention of Li-ion batteries.

Table 1 summarizes the gravimetric capacity of Li ion batteries with Ge nanoparticle anodes reported by various research groups^11,15–17^. A high gravimetric capacity over 1000 mAh/g has been achieved for 30–100 cycles. Our work is the first report on a binder-free Ge nanoparticle anode fabricated by the bottom-up plasma deposition process.

**Table 1:** Gravimetric capacity of Li ion batteries with Ge and GeSn nanoparticle anodes reported by various research groups^11,15–17^.

	Anode film type	Film fabrication process	Gravimetric capacity
Lee et al.^11^	Ge nanoparticle film	Process of binder paste	1470 mAh/g (30 cycles)
Seng et al.^16^	Ge/C nanoparticle film	Process of binder paste	1184 mAh/g (50 cycles)
Cho et al.^17^	GeSn nanoparticle film	Process of binder paste	1010 mAh/g (50 cycles)
Park et al.^15^	Ge nanoparticle assembly film	Wet etching process (binder free)	1415 mAh/g (100 cycle)
Uchida et al	GeSn nanostructure film	Plasma deposition process (binder free)	1128 mAh/g (60 cycles)

60 charging/discharging cycles is not enough to demonstrate a long life-time of nanostructured GeSn anodes. A previous study^3^ performed 350 charging/discharging cycles, for example, appropriate for the life-time required in state-of-the-art lithium-ion batteries. However, the behavior for the first 600 h of the charging/discharging cycles is important for understanding the basic characteristics of a battery electrode, as shown in the previous study^3^. Our observed 60 charging/discharging cycles roughly correspond to 600 h, and the capacity became stable after 10–20 cycles. This is valuable as the first study on nanostructured GeSe anodes produced in a single-step plasma process.

In conclusion, we fabricated highly porous amorphous Ge and GeSn nanostructured films with particle sizes of 30–40 nm by He plasma sputtering with a high He pressure of 0.1 Torr, a high gas flow rate of 200 sccm, and an off-axis substrate position of *r* = 30 mm. We evaluated the charge/discharge cycle performance of nanostructured Ge and GeSn anodes in Li-ion battery cells. A Ge anode with an arrangement of nanoparticles without aggregation showed a Li-storage capacity of 565 mAh/g after the 60th cycle and a capacity retention of 64%. The retention was markedly improved by the addition of 3at% Sn in the Ge anode. The amorphous GeSn nanostructured anode achieved a high capacity retention of 92%, and a high gravimetric capacity of 1128 mAh/g was maintained after the 60th cycle. Precise control of the nano-morphology and electrical characteristics, which is realized in a single-step procedure by using low-temperature plasma, is important for stable cycling of high-capacity Ge anodes.

## Methods

The Ge films were fabricated on a copper disk using 13.56-MHz radio-frequency (rf) magnetron sputtering, as schematically shown in Fig. 4a. The copper disk had a diameter and thickness of 15 mm and 80 μm, respectively, and was placed at the center (*r* = 0) of the substrate holder or 30 mm from the center (*r* = 30 mm), where on-axis and off-axis sputtering deposition can be performed, respectively. The sputtering target was a polycrystalline Ge disk (1-inch diameter) with a purity of 99.99%. An rf power of 60 W was supplied to the sputtering target for plasma production. He gas was supplied from the direction of the target to the substrate holder at a flow rate of 15 to 200 sccm. The He gas pressure was set to a high value of 0.1 Torr. The distance between the target and the substrate holder was 20 mm. The substrate holder was not heated or cooled during film deposition.

Ge films deposited under various He gas conditions and substrate positions were assembled into Li-ion battery cells (HS flat cell, Hohsen Corporation) as anodes with Li metal counter electrodes with diameters of 16 mm and thicknesses of 250 μm. A polypropylene separator with a diameter of 24 mm and a thickness of 24 μm was set between the Ge anodes and Li cathodes. 1-mol/L LiPF_6_ dissolved in a mixture of ethylene carbonate (EC) and diethyl carbonate (DEC) (EC:DEC = 1:1 volume%) was used as an electrolyte in the battery test cells. The battery cycle performance was analyzed at a constant current less than 1 mA, corresponding to a 0.1-C rate for all charge/discharge cycles with a cut-off voltage of 0.03–2.0 V at room temperature using a battery test system (HJ1001SD8, Hokuto Denko).

To analyze the material properties of the Ge anodes, Ge films were deposited on n-type Si wafers with a low resistivity under the same sputtering conditions as for the battery Ge anodes. The crystal structure was evaluated by Raman spectroscopy excited by 532 nm laser (T64000, HORIBA Jobin Yvon), and the surface morphology and cross-sectional microstructure were analyzed using atomic force microscopy (AFM; Dimension FastScan, Bruker) and scanning electron microscopy (SEM; SU-8010, Hitachi), respectively.
